# Ras and Raf pathways in epidermis development and carcinogenesis

**DOI:** 10.1038/sj.bjc.6606009

**Published:** 2010-11-16

**Authors:** F Kern, T Niault, M Baccarini

**Affiliations:** 1 University of Vienna, Center for Molecular Biology, Max F. Perutz Laboratories, Doktor-Bohr-Gasse 9, A-1030 Vienna, Austria

**Keywords:** Raf kinases, Ras pathway, epidermal development, carcinogenesis, differentiation therapy

## Abstract

The epidermis is the outermost layer of the body and protects it from environmental insults. This crucial function is sustained by a continuous process of self-renewal involving the carefully balanced proliferation and differentiation of progenitor cells constantly replacing the mature cells at the surface of the epidermis. Genetic changes in the signalling pathways controlling keratinocyte proliferation and differentiation disrupt this balance and lead to pathological changes including carcinogenesis. This review discusses the role of Ras, an oncogene critically involved in the development of skin neoplasia, and its downstream effector Raf in epidermal homeostasis and tumourigenesis. In particular, we will focus on the recently established role of Raf-1 as the decisive element that, by restraining keratinocyte differentiation, allows the development and maintenance of Ras-driven tumours.

The epidermis is the outer layer of the skin, a stratified squamous epithelium that forms a protective barrier against environmental insults and prevents body dehydration. The epidermis is primarily composed of keratinocytes in which pigment cells (melanocytes), immune cells (Langerhans cells and T cells) and nerve-ending cells (Merkel cells) are embedded. Invaginating into the dermis, the epidermis gives rise to various appendages: the hair, produced by the hair follicles, the sebaceous glands that lubricate the skin and the sweat glands that extrude water and salts (Figure 1A). The epidermis is separated from the dermis by the basement membrane, which is rich in extracellular matrix, and is organised into four major layers: the *stratum basale*, the *stratum spinosum*, the *stratum granulosum* and the *stratum corneum*. The *stratum basale* contains the keratinocyte progenitors required for skin renewal. These can either divide to generate further progenitor or give rise to suprabasal keratinocytes (reviewed in Blanpain and Fuchs, 2009). As they progress to the *stratum spinosum* and to the *stratum granulosum*, the keratinocytes produce the network of keratin filaments anchored in intercellular junctions that provide structural support to the skin; the cells flatten and exocytose lamellar granules containing the precursors of the lipids that contribute to the *stratum corneum* barrier. The *stratum corneum* contains the last stage of differentiation, the enucleated corneocytes, which are continuously sloughed off and must be replaced by differentiation of the lower layers (Figure 1B).

To preserve the integrity of the epidermal barrier, the epidermis turns over throughout life. Alterations in this process, such as keratinocyte proliferation/differentiation defects, or inflammation can lead to cancer or skin barrier defects; thus, the many signalling pathways involved in epidermal homeostasis must be tightly controlled.

## Ras pathways in the epidermis

**Figure 1:**
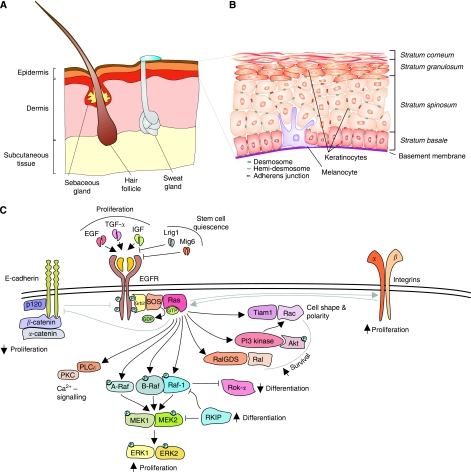
Ras pathways in the epidermis. (**A**) Schematic representation of mammalian skin. The skin consists of subcutaneous tissue, dermis and epidermis. Hair follicles and sebaceous glands invaginate into the dermis. (**B**) Structure of the epidermis. The epidermis is mainly composed of keratinocytes in various stages of differentiation. We distinguish four strata: the *stratum basale*, comprising the proliferating, undifferentiated basal keratinocytes; the *strata spinosum* and *granulosum*, containing differentiating keratinocytes; and the *stratum corneum*, with the terminally differentiated, enucleated corneocytes that are continuously removed and replaced by cells from the differentiating strata below. (**C**) The Ras pathway in the epidermis. Ras can be activated downstream of the EGFR and integrins, and can be inhibited by adhesion molecules, such as E-cadherins, inducing growth arrest. With its host of downstream effectors, Ras can mediate survival, proliferation, and can inhibit differentiation (see text for details). Arrows denote induction, blunt arrows indicate inhibition. Arrows pointing upwards signify increase, and arrows pointing downwards symbolise decrease.

The epidermal growth factor receptor (EGFR) is a critical regulator of epidermal homeostasis (Figure 1C). Binding of EGFR to its ligands, EGF, TGF-*α* and IGF, promotes keratinocyte proliferation; in contrast, inhibitory ligands of EGFR, Lrig1 and Mig6 are involved in maintaining stem cell quiescence, as ablation of either protein leads to hyperproliferation of keratinocytes (Fuchs, 2009). Downstream of EGFR, activation of Ras induces keratinocyte proliferation and inhibits differentiation (Khavari and Rinn, 2007). Ras isoforms are small G-nucleotide-binding proteins that oscillate between a GDP-bound, inactive state, and a GTP-bound state in which they can bind and activate their downstream effectors. In growth factor signalling, the transition between the inactive and the activated state is engendered by the binding of a guanine nucleotide exchange factor (GEF) that displaces GDP. Once GDP is displaced, GTP, whose intracellular concentration is ten-fold higher than that of GDP, will bind to Ras, converting it into an active form. Active Ras will then proceed to stimulate a number of effectors, including the prominent classical effectors phosphoinositide-3 kinase (PI-3K) and Raf; Ral-GDS and Tiam-1, exchange factors leading to the activation of small GTPases Ral and Rac; and several other less well-defined effectors (Figure 1C; Karnoub and Weinberg, 2008). These molecules control aspects of keratinocyte biology ranging from the regulation of proliferation, traditionally attributed to the Raf/MEK/ERK pathway (see below), or of survival, linked to the activation of PI-3K and its downstream target Akt (Sibilia *et al*, 2000; Calautti *et al*, 2005) and Ral-GDS (Gonzalez-Garcia *et al*, 2005), to the establishment of cell–cell adhesion, polarity and redox balance, controlled by Tiam1 (Mertens *et al*, 2005; Pegtel *et al*, 2007; Rygiel *et al*, 2008). Thus, activation of the Ras pathway by growth factors must be kept under tight control to provide just the right balance of proliferation/differentiation signals required for epidermal homeostasis.

Adhesion molecules, such as integrins and cadherins, have a crucial role in the modulation of growth factor signalling in the epidermis. In general, integrin activation supports proliferation, whereas cadherins can both support or inhibit it (Figure 1C); E-cadherin and desmosomal cadherins, for instance, modulate the EGF response to signal growth arrest (Muller *et al*, 2008). In line with its crucial role in proliferative signalling, the Ras pathway is activated by integrins (Janes and Watt, 2006) and negatively regulated by cell–cell adhesion: ablation of either *α*-catenin or p120-catenin in the epidermis causes a reduction of adherens junctions, which does not affect skin barrier and intercellular adhesion but leads to hyperplasia and to sustained activation of the Ras/ERK pathway (Vasioukhin *et al*, 2001; Perez-Moreno *et al*, 2006).

Activating Ras mutations stabilise this signalling-competent, GTP-bound state. These mutations are frequent in human cancer (33% higher frequency in epithelial cancers and melanoma; http://www.sanger.ac.uk/genetics/CGP/cosmic). The number of tumours (particularly squamous cell carcinomas) containing GTP-bound Ras is however much higher, likely owing to the autocrine/paracrine activation and/or to mutation in receptor tyrosine kinases such as the EGFR (Khavari and Rinn, 2007). Expression of constitutively active Ras mutants in the basal layer of mouse epidermis induces proliferation and inhibits differentiation (Vitale-Cross *et al*, 2004; Khavari and Rinn, 2007); the activation of endogenous Ras by the transgenic expression of the Ras GEFs SOS (Sibilia *et al*, 2000) and Rasgrp1 (Oki-Idouchi and Lorenzo, 2007) in the same epidermal compartment produces similar phenotypes. Consistent with an essential role of Ras in skin tumourigenesis, H-Ras ablation impairs tumour development in mouse models of chemical carcinogenesis (Ise *et al*, 2000).

The consequences of Ras activation in the epidermis can be mimicked, at least in part, by the expression of gain-of-function mutants of Ras downstream effectors. Constitutively active Akt mutants promote proliferation in skin (Murayama *et al*, 2007); conversely, the knock-in of a PI-3K mutant incapable of binding to Ras reduces tumour load in a chemical model of epidermal carcinogenesis (Gupta *et al*, 2007). Ablation of other Ras targets such as Tiam1 (Malliri *et al*, 2002) and its downstream target Rac1 (Wang *et al*, 2010), Ral-GDS (Gonzalez-Garcia *et al*, 2005) and PLCepsilon (Bai *et al*, 2004) has similar effects, but only in the case of Rac1 it is clear that the phenotype is cell autonomous.

## Raf pathways in the epidermis

The Raf/MEK/ERK cascade is the longest-studied and probably the best-described Ras effector pathway. The term ‘cascade’ already suggests the chain reaction that propels the signal, in the form of sequential phosphorylation, from an entry point to an intermediate kinase and finally to ERK, the business end of the pathway. ERK stimulation results in the phosphorylation of transcription factors, structural proteins and metabolic enzymes, and ultimately engenders both short- and long-term changes in cellular behaviour. This basic three-tiered module is robust and plastic at the same time, allowing not only the amplification but also the diversification and temporal modulation of the signal at each and every node; in mammals, it is found in four mitogen-activated protein kinase (MAPK) pathways that contribute to implementing biological outcomes as diverse as proliferation, apoptosis, differentiation, motility and response to stress and cytokines.

One outstanding feature of the Raf/MEK/ERK pathway in mammals is redundancy. Mammals have three Raf isoforms (A-Raf, B-Raf and Raf-1, also known as C-Raf), two MEK and two ERK isoforms. All three Rafs can bind to Ras and phosphorylate MEK, although B-Raf is much more efficient than the other two and is necessary for ERK activation *in vivo* (reviewed in Galabova-Kovacs *et al*, 2006a; Niault and Baccarini, 2010). Both MEKs can phosphorylate ERK1/2; and whether ERK1/2 have non-redundant functions or not is still unclear (see below).

Consistent with its prominent role as a MEK kinase, B-Raf is the component of the ERK pathway most often mutated in human tumours, with a particularly high frequency (43%) in human melanoma. The most frequent B-Raf mutation, V600E, results in constitutive catalytic activity and MEK/ERK activation. Recently, a kinase drug selectively inhibiting B-Raf V600E has achieved an unprecedented response rate of 80% in phase 1 clinical trials involving metastatic melanoma patients, opening new therapeutic avenues for this deadly disease (Bollag *et al*, 2010; Flaherty *et al*, 2010). Less frequent BRAF mutations reduce intrinsic catalytic activity but drive MEK/ERK activation by stimulating the formation of B-Raf/Raf-1 heterodimers in which mutant B-Raf activates wild-type Raf-1 in trans (reviewed in Niault and Baccarini, 2010; Wimmer and Baccarini, 2010).

Unlike B-Raf, Raf-1 is infrequently mutated in human cancer (overall frequency of 1% http://www.sanger.ac.uk/genetics/CGP/cosmic). The rare mutations detected in human cancer cell lines and in patients with therapy-related acute myeloid leukaemia do not drive tumourigenesis *per se*, but can do so upon somatic loss of a negative regulator of Raf-mediated MEK/ERK activation, the Raf kinase inhibitory protein RKIP (Niault and Baccarini, 2010). Interestingly, RKIP promotes differentiation of human keratinocytes (Yamazaki *et al*, 2004), and both RKIP and B-Raf are downregulated in human SCC (Zaravinos *et al*, 2009), whereas Raf-1 is overexpressed (Riva *et al*, 1995). Mutations or overexpression of MEK or ERK in human SCC have not been reported.

The data summarised above suggest that Raf-1 is an important player in the development of human SCC. Support for this idea came in the unexpected form of recent findings concerning small-molecule inhibitors of Raf. These compounds, some of which are already being used in the clinic, efficiently inhibit MEK/ERK activation in cells harbouring the B-Raf V600E mutation (Bollag *et al*, 2010; Flaherty *et al*, 2010), but can activate the ERK pathway to different extents in normal cells and in cells expressing Ras mutations. At the molecular level, the inhibitors promote the formation of dimers in which wild-type Raf-1 is activated in trans (reviewed in Brower, 2010; Cichowski and Janne, 2010; Wimmer and Baccarini, 2010). Before these reports, potentially deleterious effects of Raf inhibitors because of ERK inhibition in normal cells were expected to limit their clinical use. In stark contrast, these studies now predict that these inhibitors may be dangerous because they may activate Raf-1 and MEK/ERK in B-Raf wild-type cells prone to deregulated proliferation because of other mutations. Intriguingly, although the inhibitors are administered systemically, keratoacanthomas and SCC *in situ* develop in patients treated with Raf inhibitors (Brower, 2010; Cichowski and Janne, 2010; Wimmer and Baccarini, 2010), underscoring the connection between Raf-1 activation and epidermal proliferation and tumourigenesis.

The role of the Raf/MEK/ERK pathway in epidermal proliferation has been clearly established in animal models, in which inducible activation of Raf or MEK in the epidermis results in massive cutaneous hyperplasia and reduced differentiation (Khavari and Rinn, 2007). In apparent contrast, knockout of B-Raf (Galabova-Kovacs *et al*, 2006b), Raf-1 (Ehrenreiter *et al*, 2005), MEK1, MEK2 (Scholl *et al*, 2007), ERK1 and ERK2 (Dumesic *et al*, 2009) has no effect on epidermis development and/or homeostasis. In the MEK and ERK knockouts, this lack of phenotype may result from functional redundancy within the pathway. Indeed, epidermis-restricted compound MEK1/MEK2 knockout causes severe barrier function defects and marked epidermis hypoplasia leading to perinatal death (Scholl *et al*, 2007); and simultaneous ERK1/ERK2 ablation inhibits keratinocyte division (Dumesic *et al*, 2009). MEK1/2 gene dosage also appears to be the rate-limiting factor in the hyperplastic response of mouse epidermis to activated Ras (Scholl *et al*, 2009a); in contrast, however, MEK1 (but not MEK2) and ERK1 are required for full-fledged chemical carcinogenesis (Bourcier *et al*, 2006; Scholl *et al*, 2009b). Together, these results confirm the role of the ERK pathway in promoting proliferation and restraining differentiation in the epidermis.

However, recent conditional gene ablation experiments have revealed that one of the Raf kinases has ERK-independent roles in keratinocyte biology and tumourigenesis. As mentioned above, mice harbouring epidermis-restricted Raf-1 ablation do not show any major anomalies, with the exception of a curly fur and whiskers that subside after the first hair cycle. Wound healing, however, is delayed in these mice, and their keratinocytes show defects in adhesion and migration (Ehrenreiter *et al*, 2005). These phenotypes do not correlate with defects in ERK phosphorylation; rather, we have been able to trace them to the cytoskeleton-based Rho effector Rok-*α*, which is hyperactive in Raf-1-deficient cells (Ehrenreiter *et al*, 2005, 2009; Niault *et al*, 2009).

**Figure 2:**
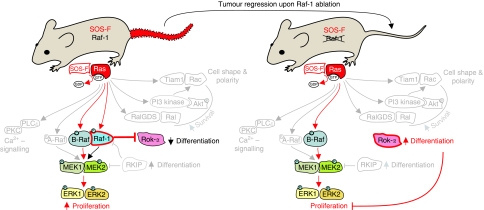
Ras-driven tumours are addicted to Raf-1. Ras-driven tumours, induced by the keratinocyte-restricted expression of membrane-tethered SOS (SOS-F transgenic mice; red, irregularly shaped tail) regress upon keratinocyte-specific ablation of Raf-1, indicating an essential role of this molecule in tumour maintenance. Downstream of Ras, Raf-1 is involved in at least two pathways: the canonical Raf/MEK/ERK pathway, where Raf-1 acts as an activator, likely in the context of a Ras-induced heterodimer with B-Raf; and the Rok-*α* pathway, where Raf-1 acts as an inhibitor via direct protein–protein interaction (left panel). Upon Raf-1 ablation (right panel; Raf-1 crossed out), ERK phosphorylation continues undisturbed, likely sustained by B-Raf; Rok-*α*, however, is strongly activated, leading to increased differentiation, and to tumour regression.

Although these data do not exclude that Raf-1 may have function(s) connected with its ability to phosphorylate MEK or other substrates, they do indicate that the essential role of Raf-1, at least in the epidermis, is independent of its kinase activity. But what happens if one stresses the system, for instance, by inducing tumourigenesis *in vivo*? We have answered this question by combining a chemical and a genetic tumourigenesis protocol with epidermis-restricted Raf-1 ablation in mice. Both models rely on the activation of Ras, caused by mutations in the DMBA/TPA chemical carcinogenesis model (Ise *et al*, 2000) or by tethering the Ras activator SOS to the membrane of basal keratinocytes (Sibilia *et al*, 2000); this results in the constitutive activation of endogenous Ras, observed more frequently than Ras mutations in human SCC (Dajee *et al*, 2003). The results were striking: Raf-1-deficient epidermis was completely refractory to tumour formation in both models; in addition, using the genetic model we could show that Raf-1 ablation enforces complete, rapid regression of established lesions that never recur, although Ras activation persists throughout the life of the animals (Figure 2; Ehrenreiter *et al*, 2009). Tumour regression is characterised by massive, runaway differentiation, which was a surprise in view of the well-established role of Raf-1 in proliferation and of its essential antiapoptotic function in other cell types (Galabova-Kovacs *et al*, 2006a). These data identify Raf-1 as the single key Ras effector absolutely required for both development and maintenance of Ras-driven tumours *in vivo*. They evoke the concept of ‘non-oncogene addiction’, intended as the absolute dependence of cancer cells on a non-mutated component of a signalling pathway to prevent system failure in the form of apoptosis or, as in this case, differentiation. Downstream of Ras, activated Raf-1 binds to Rok-*α*, reducing its activity (Figure 2); Rok-*α*, in turn, has been previously implicated in keratinocyte differentiation (McMullan *et al*, 2003) and, via the phosphorylation of Cofilin, can prevent the activation of the STAT3/Myc axis (Honma *et al*, 2006).

The relevance of the Raf-1/Rok-*α* interaction for tumourigenesis is illustrated by the fact that Ras-driven dedifferentiation, STAT3 phosphorylation and Myc expression occur both in wild-type and in Raf-1-deficient epidermis treated with a chemical Rok inhibitor (Ehrenreiter *et al*, 2009). These data establish Raf-1 as an endogenous Rok-*α* inhibitor operating downstream of Ras to regulate keratinocyte differentiation, and imply that caution is in order when using Rok inhibitors, especially in the treatment of Ras-driven tumours (Figure 2).

Rok-*α* inhibition by Raf-1 requires physical interaction between the two proteins. In growth factor-stimulated or Ras-transformed cells, the kinase domain of Rok-*α* interacts with, and is inhibited by, the regulatory domain of Raf-1, which is structurally very similar to Rok-*α*'s own autoinhibitory domain (Niault *et al*, 2009). This kind of ‘inhibition in trans’ has never been reported before, and represents a new paradigm of kinase regulation and pathway cross-talk.

Our results have implications for future therapeutic strategies targeting the Raf-1/Rok-*α* interaction, for instance, by silencing the Raf-1 gene or by using small-molecule inhibitors to disrupt the complex. The predicted outcome of such interventions is an increase in Rok-*α* activity, resulting in turn in epithelial cell differentiation. The success of these therapies, however, might be highly context dependent. We must bear in mind that Rok-*α* was found upregulated in human SSC samples, and that its overexpression confers features of malignancy to human tumours in mouse xenograft models (Lefort *et al*, 2007). It is possible that treatments aimed at increasing Rok-*α* activation might be beneficial only in the context of epithelial tumours driven by Ras activation. Alternatively, tumour cells might accumulate mutations, downstream of Rok-*α* or in other pathways, which enable them to tolerate high Rok-*α* activity without undergoing differentiation. Thus, as in the case of other approaches, the success of a Rok-*α* activation therapy might depend both on the type of tumour and on the stage of the tumour at the time of intervention.

## Conclusions

The evidence summarised above is consistent with a pivotal role of Ras and its downstream effectors in epidermal tumourigenesis. Ras effectors have been reported to induce proliferation and prevent apoptosis; recently, the differentiation block enforced by Raf-1-mediated Rok-*α* has been added to the potentially ‘druggable’ events required for Ras-driven tumourigenesis. The prediction of our *in vivo* work is that freeing Rok-*α* from its interaction with Raf-1, for instance, by silencing the Raf-1 gene or by using small-molecule inhibitors aimed to disrupt the complex, will increase Rok-*α* activity and result in epithelial cell differentiation, at least within a window of opportunity. Such a strategy should be useful in the co-therapy of Ras-driven epidermis tumours, much in the way that differentiation therapy has radically increased the success rate of leukaemia treatment (Wang and Chen, 2008).
