# Ras and Raf pathways in epidermis development and carcinogenesis

**DOI:** 10.1038/bjc.2011.108

**Published:** 2011-03-29

**Authors:** F Kern, T Niault, M Baccarini

**Correction to**: *British Journal of Cancer* (2011) **104**, 229–234; doi:10.1038/sj.bjc.6606009

Upon publication in early 2011, the authors noted the omission of an Acknowledgments section that they had intended to include. This is now included in full, below. The authors apologise for the original omission.

